# Systems immunology approaches to study T cells in health and disease

**DOI:** 10.1038/s41540-024-00446-1

**Published:** 2024-10-09

**Authors:** Aaron Yang, Amanda C. Poholek

**Affiliations:** 1 Department of Immunology, University of Pittsburgh School of Medicine, Pittsburgh, PA USA; 2 Center for Systems Immunology, University of Pittsburgh School of Medicine, Pittsburgh, PA USA

**Keywords:** Immunology, Biological techniques

## Abstract

T cells are dynamically regulated immune cells that are implicated in a variety of diseases ranging from infection, cancer and autoimmunity. Recent advancements in sequencing methods have provided valuable insights in the transcriptional and epigenetic regulation of T cells in various disease settings. In this review, we identify the key sequencing-based methods that have been applied to understand the transcriptomic and epigenomic regulation of T cells in diseases.

## Introduction

**Fig. 1:**
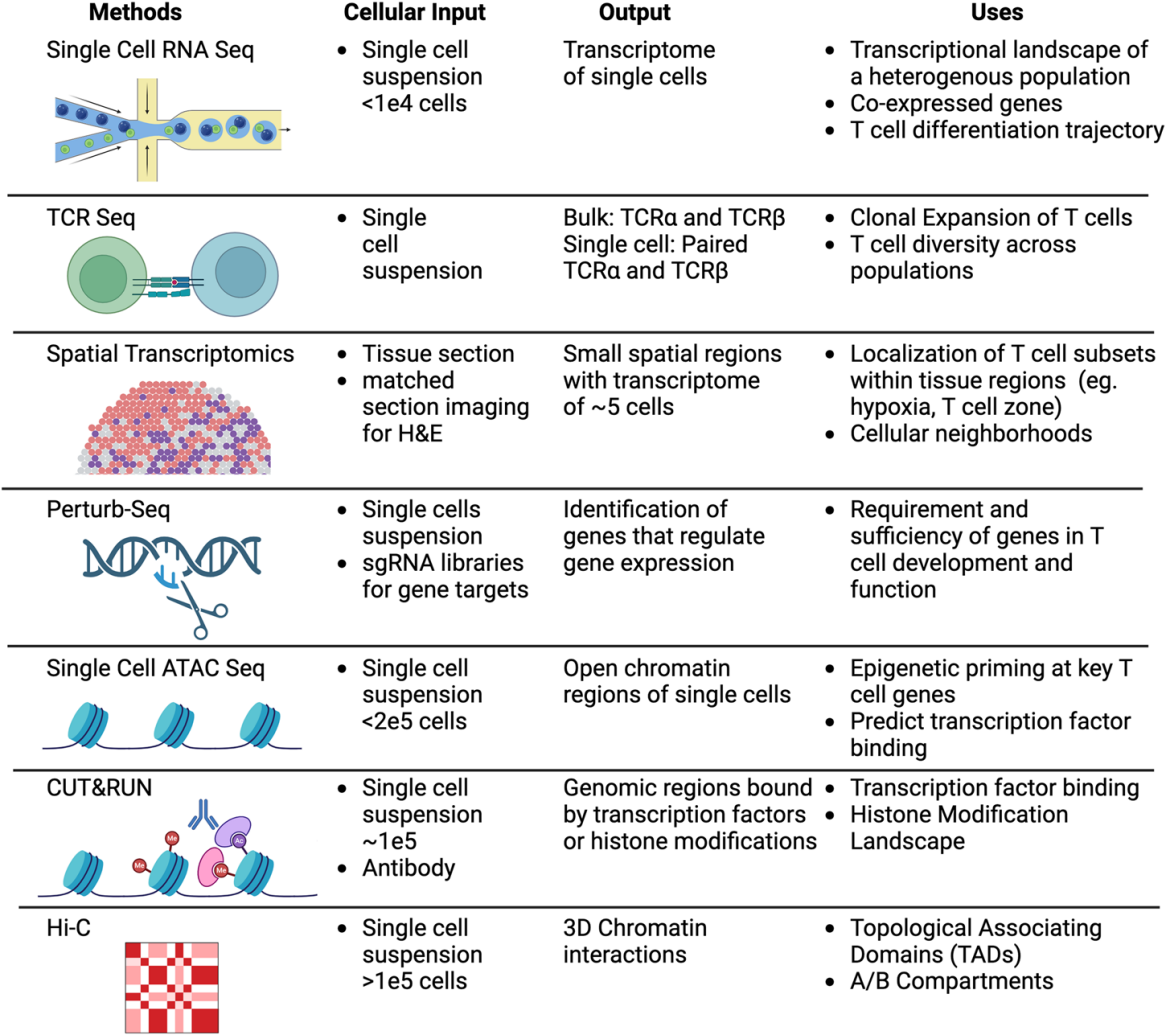
Overview of sequencing methods for investigation of T cell regulation and function. We identify cellular limitations, type of genomic output, and uses for each method. Made with Biorender.

Cost reduction and increased accessibility of next-generation sequencing (NGS) technologies has led to the rapid development of novel methods that interrogate every aspect of biology, including the epigenome and transcriptome of cells^1^. Integration of these datasets requires computational approaches that drive insight into the biology that underlies cellular interactions and function. T cells are a major player of the adaptive immune system responding to pathogens that infect the host as well as maintaining tolerance to self and innocuous environmental antigens experienced daily^2^. They are dynamic in their functional flexibility and plasticity and, therefore, must be tightly regulated to remain inactive until interaction with a pathogen, when they must rapidly expand^3^. Upon pathogen clearance, T cells must contract to prevent damaging the host with ongoing inflammation as well as retain memory T cells capable of responding to reinfection. Dysregulation of this process has been implicated in a variety of diseases ranging from cancer, autoimmunity, and viral infection. In this review, we identify key sequencing-based multi-omic methods focused on gene regulation, including transcriptomes and epigenomes, and how they reveal novel insights into the regulation of T cells in disease. Although proteomics is an emerging area with great potential to integrate with other modalities to understand T cell biology, several recent reviews have covered this topic specifically and thus will not be included here^4–6^.In each section, we provide background into the development of each technology, computational tools to interpret complex sequencing datasets, current limitations of the technology, and examples of the technology to provide novel insights into the regulation of T cells in disease (Fig. 1).

## Transcriptomics

Identifying the transcriptional landscape of cells is instrumental to understanding their regulation in a variety of disease states and tissues. Early advances in the early 2000s used microarrays to investigate RNA transcripts of nearly whole transcriptome sets of predetermined genes of interest, providing a significant understanding of T cell function^7,8^. However, the use of bulk RNA sequencing in the late 2000s and into the 2010s rapidly eclipsed microarrays due to their true unbiased ability to measure the transcriptome^9^. This method led to the discovery of novel genes that were not previously known to regulate T cell function. In this section, we will highlight recent advances in RNAseq including single-cell RNA sequencing, TCR sequencing, spatial transcriptomics and Perturb-Seq as methods to assess the diversity and function T cells.

## Single-cell RNA sequencing

Although bulk RNA-seq has been critical for our understanding of T cell populations, it requires the selection of a population of cells based on a priori knowledge of distinctions in subsets based on function or expression of surface molecules. Averaging the RNA of a bulk population limited the identification of the heterogeneity within the population. This is critical because small T cell populations are sufficient to drive disease progression, such as in tissues in autoimmunity and within tumors in cancer^2^.

Several advances allowed for single-cell assessment of the whole transcriptome of a cell. Droplet-based technologies use tissue dissociation into a single-cell suspension and microfluidics to generate oil droplets that contain a single cell with a specific sequencing barcode^10^. Other methods capture single cells into small wells, or use combinatorial barcoding throughout multiple rounds of splitting and pooling cells in bulk to achieve unique barcodes to an individual cell^11^. Collectively, these methods provide a transcriptional understanding of heterogeneous populations and dynamic cellular processes. A primary limitation of single-cell RNA sequencing (scRNA-seq) is the dropout of genes which are lower than the level of detection owing to limitations in the process of reverse transcriptase enzymes that function in small volumes^12^. This results in a significant number of genes that may be false negatives in cells with very low expression.

Previous limitations of scRNA-seq to track protein expression in conjunction with RNA expression have been overcome with the development of CITE-seq^13^ and REAP-seq^14^ that use barcoded antibodies in conjunction with droplet sequencing to infer protein expression. This provided a variety of improvements from the ability to pool several small populations together in a single scRNA-seq run where each pool is individually marked by a barcoded antibody, to the more advanced methods such as tracking adoptively transferred T cells with a distinct congenically marked protein. As more barcoded antibodies become commercially available, these methods will enhance our understanding of single-cell transcriptomes combined with protein expression.

scRNA-seq studies in T cells have made significant insights across multiple disease settings covered in previous reviews^15,16^. An early example of scRNA-seq for the investigation of T cells was in metastatic melanoma patients to identify cellular environments within the tumor. Prior to scRNA-seq, bulk RNA-seq data from metastatic melanoma samples found that tumor-infiltrating lymphocytes (TIL) have exhaustion signatures and expression of inhibitory receptors^15–17^. Using scRNA-seq, Tirosh et al. investigate the relationships between exhausted T cell signatures with other cell types within the tumor^18^. Specifically, cancer-associated fibroblasts (CAF) had specific gene signatures associated with T cell infiltration suggesting key interactions between these cell types. Furthermore, they identify novel genes of T cell exhaustion by identifying cells that expressed inhibitory receptors which are classical markers of exhaustion. scRNA-seq provides the methodology to correlate transcriptional signatures of one population to another to infer cellular interactions. Further, it allows for the clustering of gene signatures to identify novel genes associated with specific T cell types.

Another study of T cells in the tumor microenvironment leveraged scRNA-seq to identify novel T cell differentiation trajectories within the tumor. Prior to scRNA-seq, it was difficult to identify intricacies of gradual differentiation within a heterogeneous population. Zheng et al. use scRNA to track the differentiation trajectory of T cell populations in the tumor^19^. This revealed that exhausted CD4 and CD8 T cells were similar to an intermediate population of effector T cells, which are promising targets for immunotherapy. They identify similar differentiation patterns between exhausted CD4 helper T cells and Treg suppressive cells, but do not share TCR clonality, suggesting that there are independent factors that drive these cell populations.

Due to the high dimensional nature of scRNA-seq with thousands of genes within thousands of cells, the development of bioinformatics tools for scRNA-seq data analysis has been critical for the interrogation of datasets. Basic analysis for scRNA-seq using pipelines, such as Seurat^20^, clusters single cells by similarity and assigning annotations to each cluster. However, rare cell populations remain challenging to accurately annotate due to limitations in curated marker gene lists, transitory states of cells or technical issues in batching^21^. Machine learning approaches to improve annotation, such as scBERT^22^ and devCellPy^23^, improve upon the annotation of clusters to gain a better understanding of individual cells. scBERT generates a deep neural net training model that learns gene–gene interactions without annotation from large scRNA-seq datasets followed by supervised annotation. In contrast, devCellPy adds the element of time to highly curated and annotated reference datasets to improve the annotation of transitory cell states, improving the resolution of annotation in a complex and dynamic system. Advanced exploratory analysis has also been critical for advancing our understanding of T cells. SCENIC^24^ and the improved SCENIC+^25^ was developed as a computational gene regulatory network to infer transcription factors enriched in cell clusters and has been used for example, to identify TF regulons associated with terminal exhaustion of T cells in tumors^26^. This previously could not have been achieved on a bulk RNA-seq level or experimentally devised to the diversity of TF binding and cellular heterogeneity. Another key bioinformatic tool for understanding dynamic systems such as T cell differentiation are pseudo-time analysis and RNA velocity methods to infer the differentiation trajectory of T cells^27^. Monocle3^28^, scVelo^29^, and the recently developed TopicVelo^30^ and TFVelo^31^ are all methods that aim to better resolve our understanding of cellular differentiation using the single-cell space. While each method takes a slightly different computational approach, these methods have been highly valuable for understanding complex cell trajectories and inferring transitory states as cells move from one state to another. For T cells, this is crucial, as we know a small number of naïve T cells in a pool can recognize peptide antigen and respond by differentiating into one of a number of possible states. For example, monocle has been used to identify CD4+ effector gradients of resting T cells^32^, and to understand the complex transitory states that CD8 T cells take during acute or chronic viral infection^33^. Collectively, these analyses revealed many more transitory T cell states than possible to identify by traditional methods or even bulk RNAseq with complex cell sorting schemes. Combining powerful computation approaches with technological advances has reframed our understanding of T cell differentiation and provided insight into the dynamics of T cell differentiation that was previously unrecognized.

scRNA-seq has provided a significant technological advancement to improve the understanding of dynamic and heterogeneous T cell populations within disease environments. Furthermore, the droplet-based technology for isolation and sequencing of individual cells has been applied for advancements in single-cell ATAC-seq and single-cell CUT&RUN to provide a more comprehensive understanding of T cell biology, as discussed below.

## TCR sequencing

T cells are unique in that each cell has a unique T cell receptor (TCR) that can recognize presented antigens. A functional TCR comprises two proteins, an alpha and a beta chain. T cells recognize a massive number of antigens due to the process of VDJ recombination, where T cells randomly select unique combinations of TCR sequences^34^. Upon T cell recognition of cognate antigen, they quickly undergo rapid proliferation where the TCR sequence is retained to recognize the same antigen. Thus, the TCR sequence acts as a “natural” barcode that can track daughter cells coming from a specific T cell. Tracking TCR receptors within a bulk population, and, importantly, linking that information to cellular differentiation in an expanding population has been previously limited to a priori knowledge of a specific immunodominant antigen for which tetramers that bind a TCR can be generated, or a specific TCR sequence clone is generated as a transgenic TCR. Importantly, TCR-seq can be combined with scRNA-seq to identify a pattern of TCR sequences and the functional diversity within a population of T cells^35^.

Early TCR sequencing methods were limited by their ability to capture only partial sequences of specific V-J regions^36^. Bulk TCR sequencing provides a high depth of sequencing and is useful for approximating the diversity of the TCR sequences between populations. However, bulk TCR sequencing cannot resolve the pairing of TCRα and TCRβ chains at the level of the individual T cell. This limitation prevents accurate estimations of clonotype diversity as different TCRα may pair with multiple TCRβ chains. Further, this prevents a full understanding of TCRs because the combined pair is critical for antigen specificity^36^. Thus, the advent of single-cell methods provided major advances in segregating specific TCRαβ pairs for the identification of TCR clonotypes of an individual T cell. The integration of transcriptional single-cell RNA sequencing and TCR sequencing further provides insights into T cell clonal expansion and T cell antigen specificity. A current limitation, in addition to cost considerations, is the low efficiency of single-cell TCR sequencing (approximately 65%), which prevents the identification of rare T cell populations^36^. Improving the efficiency rate will improve the accuracy of the identification of clonotypes.

TCR sequencing has provided novel insights into T cell function in the context of a variety of diseases. Prior to TCR sequencing, it was known that T cells target cancer antigens, but the characteristics of specific T cells that target cancer epitopes was not well understood. He et al.^37^ used TCR sequencing to identify T cells recognizing neoantigens, which are mutated antigens that are uniquely found in tumor cells. Using paired TCR-sequencing and scRNA-seq, this study identified that T cells specific to tumor neoantigens express the marker CXCL13 predicted in response to checkpoint inhibitor therapy, as well as elucidating the critical role of neoantigen specific T cells.

In the context of COVID-19, Schultheiβ ^38^ characterized T cell clones specific to SARS-CoV-2 from patients with active infection or after recovery. Using bulk TCR sequencing from patients T cells, they identified SARS-CoV-2 specific T cell clusters were associated with increased disease severity. This study highlights the importance of T cell expansion and contraction in response to pathogens which can be used to improve vaccine development.

The “holy grail” of TCR analysis is to understand which TCR sequence can recognize a given peptide sequence derived from an antigen. While we remain a long way off from knowing the peptide that binds to a TCR based on its sequence, major strides in this area have been achieved from scTCR-seq data and computational methods that can relate clonality. Several methods aim to close this gap, such as GLIPH2^39^, TCRdist^40^, or GIANA^41^ that group TCRs based on TCR sequence similarities or differences. DeepTCR^42^, VDJdb^43^, and others aim to match predicted TCRs to known epitope interactions. Finally, Cytoscape^44^ can provide visualization of the TCR repertoire by indicating similarities between clonotypes and representing the TCR repertoire as a network. These methods have been described in detail in the following reviews: Valkeirs et al.^45^, Brown et al.^46^, and Bradley et al.^47^. While new technologies will likely need to be combined with computational approaches to achieve the prediction of epitopes from the TCR sequence alone, current approaches have already vastly improved our understanding of T cell recognition of antigens from scTCR-seq and computational approaches.

## Spatial transcriptomics

As previously described, scRNA-seq has been incredibly valuable for understanding dynamic and heterogeneous cell populations. However, scRNA-seq requires cells to be isolated from dissociated tissues, thus eliminating the spatial context in tissues of cell-cell interaction. In the context of T cells, this is particularly important as T cells traffic into tissues and localize at important sites to fight infection and cancer, but also can localize to tissues to drive autoimmunity. The loss of this spatial information prevents researchers from understanding the localization of each cell in areas of tissues or proximity to other cell types. To overcome this limitation, the development of spatial transcriptomics has provided several ways to identify the transcriptome of the cell and simultaneously identify its cellular localization in situ^48^. There have been several platforms developed, some of which are commercially available, that each have key strengths and weaknesses.

Broadly, spatial transcriptomics can be split into two distinct methods: Image-based technologies and Sequencing-based technologies. The first method uses probes specific to mRNA that can be multiplexed using serial rounds of imaging. Fluorescence in situ hybridization (FISH) of specific oligonucleotides for mRNA transcripts are hybridized to tissues that have been traditionally cut onto glass slides. Using various methods such as on-slide amplification, branched chain hybridization, or combinatorial barcoding, fluorescent tags are bound in small sets followed by imaging. The fluorescent tags are then quenched, and a new round of tags are added. Sequential rounds of imaging can allow for thousands of targeted probes to be detected in a single tissue slice. Critically, these methods capture mRNA information at the single cell or subcellular level, providing high-resolution information. There are several commercial platforms using Image-based technologies available that each requires the purchase of an instrument (Vizgen MERSCOPE, Nanostring CosMx, and 10X Genomics Xenium). The use of probe-based strategies can limit the number of RNAs detectable on a single sample and the species availability (typically only human and mouse), but is an advantage for detection of microbial or viral co-detection in a tissue space, which is an area ripe for discovery.

In contrast, sequencing-based methods allow for unbiased capture of RNA in tissue using barcoded probes immobilized on glass slides (10X Genomics Visium) or nanobeads layered onto slides (Slide-seq^49^). Barcoded probes contain poly-dT segments capable of capturing RNA that diffuses from tissue upon permeabilization. These methods are usually not single-cell resolution, however newer versions with increased numbers of barcoded regions (VisiumHD) or photocleavable linkers on beads that are diffable into nuclei for single nucleus labeling (Slide-tags) hold promise for sequencing-based single-cell resolution. Regardless, sequencing-based technologies do not require the purchase of a large instrument, provide unbiased gene level detection, and is cross species-applicable, making this option advantageous in some areas compared to imaging-based technologies. The specifics of spatial technologies have been covered in other reviews^50^.

As this is still an area of rapid development, spatial transcriptomics stands to revolutionize our understanding of in situ tissue biology, which has several areas of interest for T cell biologists. Nirmal et al.^51^ used spatial transcriptomics to investigate immune regulation in cutaneous melanoma, a highly immunogenic tumor. Using spatial transcriptomics, they find the interaction between tumor cells and activated PD-1^+^ cytolytic T cells are in close proximity, but nearby suppressive T_regs_ and PD-L1^+^ myeloid cells create a suppressive environment that inhibits cytolytic T cells from killing tumor cells, promoting tumor persistence. The spatial proximity of PD-1^+^ T cells and PD-L1^+^ myeloid cells provides novel insights to the mechanisms of T cell suppression in the tumor microenvironment. To improve the capabilities of spatial transcriptomics, Liu et al.^52^ developed a method to combine spatial transcriptomics and TCR-seq called Slide-TCR-seq. An outstanding question in the field was how TCR clonotypes specific to tumors are localized within the tumor microenvironment. Using Slide-TCR-seq, they identify distinct clones within renal cell carcinoma and melanoma to understand how localization may affect therapeutic efficacy or resistance. Expanded TCR clonotypes after checkpoint inhibitor therapy were found more frequently within the tumor compared to normal tissue, and T cells found deeper in the tumor exhibited exhaustion transcriptomes and poor response to checkpoint inhibitor therapy. This study provided insights into T cell localization in the tumor correlating with their function.

Computational approaches for spatial transcriptomics are an area that is currently in rapid development at the writing of this review. While many current methods leverage single-cell approaches, many are aiming to develop approaches needed specifically for spatial transcriptomics. There are several computational challenges. The first is the deconvolution of transcriptomes to individual cells. Although theoretically not needed if technology is at the single-cell level, cells in tissues are not uniform and do not line up accordingly to the barcoded spaces arrayed on any current technology. For imaging-based techniques, cell morphological stains are needed to identify individual cells to know which transcript belongs to which cell. These are separate computational challenges based on the technology used. As T cells tend to be small when quiescent (naïve or memory) and significantly larger when blasting or in an effector state, these challenges are increased when trying to identify individual T cells in a tissue space. Understanding functional niches within tissues requires further computational approaches. Cell-cell interaction algorithms such as CellphoneDB^53^, CellChat^54^, or CellTalker^55^ developed for single-cell RNAseq can be leveraged, but secreted products that locally interact in an environment are a key function of T cells. Thus, using approaches that identify cells expressing a cytokine for example, while locally identifying cells that respond to that cytokine, either through expression of the ligand and expression of target genes downstream of that cytokine-signaling interaction would be a powerful tool for understanding T cell function in the tissue space. As this is a newer technology, computational approaches that fully leverage the advance are yet to be developed, however there is no question there is much to learn about T cell function and differentiation when applied to the tissue space.

## Perturb-seq

Understanding the function of specific genes and their relationship to phenotype remains an open question across many fields. For T cells, genes can be deleted in mouse models using a Cre-Lox system for T cell- specific deletion to understand their function^56^. However, this method can be time-consuming and expensive. With the development of genetic editing via CRISPR, Perturb-seq was developed that leveraged CRISPR technology with single-cell RNA sequencing to screen genetic perturbations at a massive scale while monitoring effects at the single-cell level^57,58^. Pooled CRISPR sgRNA libraries that inactivate genes either by dsDNA breaks or inactivating transcription (CRISPRi) are transduced along with Cas9 into cells, followed by scRNA-seq and analysis of transcriptional changes related to specific gene perturbation.

Perturb-seq has been used to interrogate specific pathways of T cell function. Belk et al.^59^ use a genome-wide CRISPR screen to investigate chromatin remodeling factors and their role in T cell exhaustion. They identify that T cells within the tumor are regulated by the INO80 and BAF transcription factor complexes that limit T cell persistence within the tumor. In another study, Larson et al.^60^ used Perturb-seq to assess the efficacy of chimeric antigen receptor (CAR) T cell therapy against solid tumors. To identify resistance mechanisms that prevent CAR-T cell function, they used a genome-wide CRISPR Perturb-seq in a glioblastoma tumor model and found that loss of interferon-γ receptor genes improved CAR-T efficacy specifically in solid tumors. These findings can improve T cell engineering strategies to improve cancer outcomes. Perturb-seq is a useful method to identify the sufficiency and necessity of specific genes in T cell differentiation and function at a high throughput scale by leveraging scRNA-seq technology. However, given the number of genes available to perturb, the cost and scalability of Perturb-seq remains a challenge. To overcome this, a recent update called compressed Perturb-seq which develops algorithms that can interpret multiple random perturbations per cell or multiple cells per droplet by leveraging the sparse structure of regulatory circuits^61^. This method leverages the sparsity of perturbation effects to learn the individual effect of any one perturbation by measuring a combination of random perturbations (termed composite samples) and extract the effect of the individual perturbation using sparsity-promoting algorithms. This method will allow for greater numbers of perturbations to be performed while limiting the scale and cost, achieving greater power of this method.

## Epigenomics

Epigenetics are the study of heritable changes that alter cellular function without changing the DNA sequences of cells. Importantly, epigenetic modifications are inherited across cell division and thus play an important role in T cell differentiation and cell type-specific expression of genes^62^. Similar to transcriptomics, the advent of NGS has improved many epigenetic methods to better understand the dynamic regulation of T cells and function. In this section, we will describe two primary methods to investigate the epigenome of T cells through chromatin accessibility and histone modifications and their relationship to transcriptomics. Further, alterations to the epigenome can impact the structure of chromatin and chromatin interactions that regulate cell states and function.

## Single-cell ATAC sequencing

Chromatin accessibility is a critical part of gene regulation. Though several methods have been used to measure open chromatin regions (DNAseq^63^, FAIREseq^64^), the most recently heavily used method for T cells is ATAC-seq (assay for transposase-accessible chromatin)^65^, which uses a hyperactive Tn5 transposase to identify open chromatin regions. ATAC-seq can be performed on small cell numbers making it particularly beneficial to studying T cells ex vivo in disease settings or specific tissues. Although much information has been gained from purified populations of cells in bulk, in 2019, single-cell ATAC-seq (scATAC-seq) was developed by applying droplet-based barcoding to ATAC-seq^66^. These methods allow for the identification of alterations of open chromatin regions of rare and dynamic cell populations for up to 2 × 10^5^ cells^67^.

Recent advances in computational methods have improved upon ATAC-seq data to use open chromatin regions to infer transcription factor activity. ChromVar^68^, TOBIAS^69^, and the recently developed ChromBPnet^70^ use bulk ATAC-seq datasets and correct enzyme bias to extract the specific peak footprints within open chromatin that are left by transcription factor binding. Rather than predict the likelihood of binding based on TF motifs with Homer^71^, these methods can infer the binding of TFs at specific sites. CistromeDB^72^ is a useful accompanying method to ChromBPnet which uses publicly available ChIP-seq data to identify binding of specific TFs to genomic regions. These methods provide a computational method to discover and rank top candidate transcription factors on T cell states allowing for more efficient cellular validation.

ATAC-seq and scATAC-seq have been used to understand T cell function in a variety of diseases. Scott-Browne et al.^73^ uses bulk ATAC-seq to investigate how naïve CD8^+^ T cells respond to viral infection as they progress from naïve T cells, activated effector cells and long lived memory cells. They find each CD8^+^ T cell population is epigenetically distinct with open chromatin regions at cell-specific genes with predicted binding motifs of cell type-specific transcription factors. Further, T cells that cannot clear a chronic viral infection become exhausted with decreased function but retain open chromatin region landscape to effector T cells suggesting that they are epigenetically primed to become more functional.

Satpathy et al.^66^ further investigates epigenetic heterogeneity of T cell exhaustion in the context of cancer. They apply scATAC-seq to patient samples of blood and basal cell carcinoma who are undergoing checkpoint inhibitor therapy. Using this method, they identify cell type-specific regulatory elements within a diverse group of cells that control T cell exhaustion. They find that a group of T cells that are responsive to checkpoint inhibitor therapy share genes and transcription factor binding motifs between exhausted CD8^+^ T cells and CD4 T_FH_ cells which provides novel insights into the understanding of the mechanisms of checkpoint inhibitor therapies.

## CUT&RUN

Profiling epigenetic features such as transcription factor binding and histone modifications are important for understanding the epigenetic landscape of T cells. Identifying the location of transcription factor binding provides a broad understanding of the regulatory functions of transcription factors at specific genes. ChIP-seq has been the standard method, which uses antibodies for a target protein, sonication to shear chromatin, and finally, immunoprecipitation to isolate DNA sequences bound by the target protein^74^. However, it was difficult to perform on T cells in disease contexts since ChIP-seq requires at least 1 × 10^6^ cells. Thus, there has been a lack of understanding of the binding of transcription factors and epigenetic features from ex vivo T cells in cancer and other disease settings.

CUT&RUN was developed by Steve Henikoff in 2017^75^. Similar to ChIP-Seq, it uses an antibody specific to transcription factors or histone modifications to pull down regions of interest. Unlike ChIP-seq which requires DNA sonication, CUT&RUN uses the conjugate protein pAG-MNase that binds to the antibody and cuts DNA at nearby regions which prevents loss of DNA. This decreases the minimum cellular input from 1 × 10^6^ cells of ChIP-seq to a few thousand cells for CUT&RUN. Further improvements of the method in the last few years include CUT&Tag^76^ which is a streamlined protocol for targeting histone modifications and TFs that bind strongly to DNA. CUT&RUN remains preferable for TFs that are more loosely bound to DNA. Lastly, there has been developments in single-cell CUT&Tag to investigate histone modification profiles at a single-cell resolution^77^, which can improve the investigation of dynamic and heterogenous populations.

Development of single-cell CUT&Tag^77^ overcomes challenges to understand transcription factor binding on a per cell basis which can identify alternations in binding of cells within a heterogenous population. As with all antibody-based assays, the assay is also limited by the specificity and avidity of the antibody. Several transcription factors lack reliable antibodies which prevents the investigation of the genomic binding of these transcription factors.

CUT&RUN and CUT&Tag datasets provide genome-wide peaks for histone modifications and TFs, but how these peaks relate to cell states requires computational analysis. Computational approaches to provide functional analysis CUT&RUN datasets with motif analysis to estimate the likelihood of transcription factor binding to estimate how TFs regulate enhancer sites^78^. Methods such as ChromHMM^79^ or Segway2.0^80^ assign chromatin states using the whole genome to create chromatin state annotation segregate histone modification and transcriptional states. This provides which can be applied to understand varied T cell states from genome wide datasets^81^.

CUT&RUN and CUT&Tag have been used to investigate transcription factor regulation of T cell function as well as histone modifications that epigenetically regulate T cell function and differentiation. Mitchell et al.^82^ use CUT&RUN and CUT&Tag to investigate the role of H3K27me3 (repressed chromatin) and the histone demethylase UTX during T cell response to viral infection. Since CUT&RUN and CUT&Tag is a genome-wide assay, they profile how UTX deficiency in T cells impacts H3K27me3. They find that UTX has a critical role in demethylating H3K27me3 at genes of effector molecules for increased antiviral activity, as well as increasing inhibitory receptor activity to reduce T cell longevity. The combination of CUT&RUN and CUT&Tag improves upon the understanding of the epigenetic regulation of T cell activation and contraction.

To investigate the epigenetic regulation of exhausted T cells, work from our group^81^ used CUT&RUN to profile histone modifications of exhausted CD8^+^ T cells in the tumor as they progress from functional progenitor exhausted cells to dysfunctional terminally exhausted cells. We use CUT&RUN to profile active histone modifications such as active enhancers, active promoters, and repressive marks. Using this data set, we identify novel epigenetic features of terminally exhausted cells that have increased regions of histone bivalency with both active and repressed histone modification as well as regions of active histone modifications without corresponding RNAs. This provides novel insights into how histone modification landscape impacts T cell function and drives T cell dysfunction.

## 3D chromatin interactions

3D chromatin interactions are important for understanding the underlying mechanisms that regulate epigenetic and transcriptional landscapes of cell types^83^. Hi-C has been the most widely used for 3D chromatin studies. This assay cross-links chromatin, ligates regions in close proximity followed by deep sequencing. Hi-C identifies all regions that are interacting rather than a targeted region. This generates -omics level datasets that capture the 3D chromatin topology of cell types. Broad 3D chromatin topology has yielded an understanding of higher-order chromatin features, such as topological associating domains (TADs) that have gene regulatory functions^84^. There have been developments in Hi-C in recent years with single-cell Hi-C to overcome heterogeneous datasets from bulk Hi-C^85^. Understanding 3D chromatin topology improves upon the understanding of the regulation of T cell function beyond transcriptional factor activity and epigenetic mechanisms. Since these data contain several hierarchical orders of chromatin structures, bioinformatic pipelines are critical to identify TADs, chromatin interactions, and promoter–enhancer interactions which have been compared in this review^86^. There have also been computational methods to improve upon Hi-C data calling. One common limitation of Hi-C is the low resolution of the datasets. A computation prediction model, DeepLoop^87^, enhances the resolution of Hi-C by using deep-learning to perform bias-correction to map chromatin loops from even single-cell Hi-C.

Hi-C requires a high (>1e6) number of cells for input into the Hi-C assay and is a low-resolution method for resolution at kilobase distances^88^. To overcome these limitations, assays have been developed to improve upon the cellular input and increase the sensitivity. Examples of these assays are Hi-TRAC^89^ and Hi-CAR^90^, many of which have not yet been applied to investigate the role of T cells in disease settings. Further, there have been advances, such as Hi-ChIP, which combines ChIP-seq methods to Hi-C to better profile specific regions (e.g., Enhancers)^91^.

Hi-C has been applied to understand the 3D chromatin regulation of T cells in differentiation and disease. Russ et al.^92^ use Hi-C to investigate how 3D chromatin of naïve CD8^+^ T cells differentiation in response to infection. They find T cell states have specific 3D genome configuration and that the BACH2 transcription factor restrains the naïve T cell state by enforcing the chromatin state. In another study, Kloetgen et al.^93^ use Hi-C to assess how T cell acute lymphoblastic leukemia (T-ALL) have altered chromatin architecture in response to small molecule drugs. They initially find that T-ALL have distinct 3D chromatin with alterations of TAD boundaries at the Myc locus, which drives cellular proliferation. They find that in response to small molecule drugs, there are alterations in 3D chromatin interaction at these sites. These data highlight the need to investigate small molecule drugs that target 3D chromatin structures as potential for drug development to improve T cell function.

## Conclusions

In the last 10–15 years a significant number of NGS technologies have been developed that both increase our ability to assess the cellular state at the single-cell level as well as understand both the transcriptional and epigenetic state of cells. Single-cell and low-input approaches have been particularly useful for T cell biology leading to significant advances in our understanding due to both the tremendous heterogeneity of T cells, as well as the presence of a small number of cells to have impacts on disease states. Finally, as primary cells from ex vivo states are often harder to work with, advances in NGS approaches have been instrumental for T cells, which are frequently studied in vivo and thus not readily useful for more traditional approaches. The continued development of multi-omic methods and integration of sequencing -omics to proteomics and metabolomics will provide a novel understanding of T cells. There is no question that new technological advances in this area will only further increase our knowledge of T cells in many key settings of disease.
